# EWF: simulating exact paths of the Wright–Fisher diffusion

**DOI:** 10.1093/bioinformatics/btad017

**Published:** 2023-01-11

**Authors:** Jaromir Sant, Paul A Jenkins, Jere Koskela, Dario Spanò

**Affiliations:** Department of Statistics, University of Warwick, Coventry CV4 7AL, UK; Department of Statistics, University of Warwick, Coventry CV4 7AL, UK; Department of Computer Science, University of Warwick, Coventry CV4 7AL, UK; The Alan Turing Institute, British Library, London NW1 2DB, UK; Department of Statistics, University of Warwick, Coventry CV4 7AL, UK; Department of Statistics, University of Warwick, Coventry CV4 7AL, UK

## Abstract

**Motivation:**

The Wright–Fisher diffusion is important in population genetics in modelling the evolution of allele frequencies over time subject to the influence of biological phenomena such as selection, mutation and genetic drift. Simulating the paths of the process is challenging due to the form of the transition density. We present EWF, a robust and efficient sampler which returns exact draws for the diffusion and diffusion bridge processes, accounting for general models of selection including those with frequency dependence.

**Results:**

Given a configuration of selection, mutation and endpoints, EWF returns draws at the requested sampling times from the law of the corresponding Wright–Fisher process. Output was validated by comparison to approximations of the transition density via the Kolmogorov–Smirnov test and QQ plots.

**Availability and implementation:**

All softwares are available at https://github.com/JaroSant/EWF.

**Supplementary information:**

Supplementary data are available at *Bioinformatics* online.

## 1 Introduction

The Wright–Fisher diffusion is a central model for the temporal fluctuation of allele frequencies in a large population evolving under random mating and in the presence of mutation and selection. Despite its importance, it remains difficult to work with from a computational perspective, both in the absence of selection (where the transition density admits an infinite series expansion) and the non-neutral case (where the corresponding infinite series expansion has intractable terms). Additionally, in a diallelic model, the diffusion lives on the bounded interval *[*0, 1*]*, and thus even simple approximate sampling techniques such as the Euler–Maruyama scheme require sophisticated modifications to respect its boundary behaviour (Dangerfield *et al.*, 2012). Existing approaches in the literature have tackled this by resorting to a combination of discretization and numerical approximation, e.g. solving the Kolmogorov backwards equation numerically (Bollback *et al.*, 2008; Malaspinas *et al.*, 2012), approximating through more tractable processes (Mathieson and McVean, 2013), truncating a spectral expansion of the transition density (Steinrücken *et al.*, 2016) and using Riemann sum approximations (Schraiber *et al.*, 2016), all of which induce a bias which is hard to quantify.

In some cases, *exact* sampling routines making use of rejection sampling are available. This class of techniques has been extended to certain variants of the Wright–Fisher diffusion: Jenkins and Spanò (2017) showed that neutral Wright–Fisher diffusion paths and bridges can be simulated exactly via simulation techniques tailored for infinite series, and that neutral paths are the natural proposal mechanism for simulating non-neutral paths by rejection. Their work assumes that the mutation parameters are strictly positive and the endpoints for both the diffusion and diffusion bridge lie in the interior of *[*0, 1*]*. The case of diffusion bridges that start and end at 0 was tackled by Griffiths *et al.* (2018), but several other combinations of startpoint, endpoint and parameters remain unaddressed. Moreover, no simulation package implementing all of the cases of interest exists.

We present EWF, a C++ package producing exact draws from both neutral and non-neutral Wright–Fisher diffusions. The method properly accounts for all types of boundaries (entrance, reflecting and absorbing), incorporates a wide class of selection models and allows for arbitrary endpoints, substantially extending previous work by Jenkins and Spanò (2017) and Griffiths *et al.* (2018). These new theoretical details can be found in the accompanying supplement. Additionally, EWF preserves accuracy over long times, in contrast to Euler–Maruyama type schemes where errors accumulate over the simulated path.

## 2 Models

Consider the two-allele non-neutral Wright–Fisher diffusion ${\left(X_{t}\right)}_{t\geq 0}$ with mutation parameter $\theta =(\theta_{1},\theta_{2})$, which is given by the solution to the following stochastic differential equation

(1) $$\begin{array}{c} dX_{t}=\frac{1}{2}\left[\sigma X_{t}(1-X_{t})\eta (X_{t})-\theta_{2}X_{t}+\theta_{1}(1-X_{t})\right]dt\\ +\sqrt{X_{t}(1-X_{t})}dW_{t} \end{array}$$

for t≥0 with $X_{0}\in [0,1]$, and $\eta (x)=\sum_{i=0}^{n}a_{i}x^{i}$ for *n* finite (e.g. for genic selection η(x)=1 and for diploid selection η(x)=h+x(1−2h) with *h* the dominance parameter). When the mutation parameter θ has positive entries, the corresponding neutral (i.e. σ=0) transition density can be decomposed into a mixture distribution

$$\begin{array}{c} p^{\left(\theta_{1},\theta_{2}\right)}(x,y;t)=\sum_{m=0}^{\infty }q_{m}^{\theta }(t)\sum_{l=0}^{m}{\text{Bin}}_{m,x}(l){\text{Beta}}_{\theta_{1}+l,\theta_{2}+m-l}(y), \end{array}$$

where ${\left(q_{m}^{\theta }(t)\right)}_{m\in N}$ is a distribution on the integers and $\theta :=\theta_{1}+\theta_{2}$. This allows for exact simulation (Jenkins and Spanò, 2017, Section 2). EWF extends this approach to the $\theta_{1}=0$ and/or $\theta_{2}=0$ cases, when the diffusion is absorbed on hitting 0 and/or 1 in finite time almost surely.

It is often of interest to consider the evolution of a de novo mutation which appears at time $t_{0}$ and is observed in the population at a sampling time $t>t_{0}$. If θ=0, one needs to condition the diffusion on non-absorption to recover a non-degenerate transition density. The resulting density can be found in Supplementary Information Section S1 (together with the respective details), as well as the corresponding transition densities for the cases when θ=(0,θ) or θ=(θ,0).

The transition density for a diffusion *bridge* can be similarly derived (see Supplementary Information Section S2), whilst in the presence of selection [i.e. σ≠0 in (2)], draws from the corresponding non-neutral process can be returned by simulating neutral paths as candidates in an appropriate rejection scheme (Jenkins and Spanò, 2017, Section 5).

## 3 Methods

The expression for $p^{\left(\theta_{1},\theta_{2}\right)}(x,y;t)$ tells us that draws from the transition density can be achieved by the following:

1. Draw $M∼{\left\{q_{m}^{\theta }(t)\right\}}_{m\in N}$
2. Conditional on M=m, draw L∼Bin(m,x)
3. Conditional on M=m,L=l, draw $Y∼\text{Beta}(\theta_{1}+l,\theta_{2}+m-l)$

Steps 2 and 3 are simple. Step 1 is more involved since each $q_{m}^{\theta }(t)$ is an infinite series (see Supplementary Information Section S3 where we have extended the procedure to generate samples when θ=0 or θ=(0,θ)).

If the time increment *t* is small, approximations are necessary due to numerical instabilities in computing $q_{m}^{\theta }(t)$. EWF employs a Gaussian approximation of $q_{m}^{\theta }(t)$ for small *t* (Griffiths, 1984, Theorem 4) (t≤0.08 by default), with similar approximations used for bridges whenever subsequent time increments fall below some threshold. For full details see Supplementary Information Section S5.

**Fig. 1.**
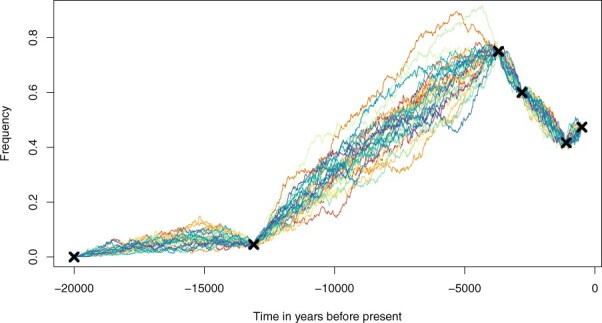
Illustration of 30 candidate trajectories for the horse coat colour data found in Ludwig *et al.* (2009) simulated using EWF (note that the observed frequencies (black crosses) are assumed to be exact observations of the underlying diffusion). Simulations used the inferred selection coefficient s=0.0007 with a consensus effective population size *N*_e_ = 10 000 (Ludwig *et al.*, 2009; Malaspinas *et al.*, 2012; Schraiber *et al.*, 2016), giving $\sigma =2N_{e}s=14$. We used θ=0 and a generation time of 5 years

The implementation was tested extensively and validated through a combination of QQ plots and the Kolmogorov–Smirnov test (see Supplementary Information Section S7). An example is shown in Figure 1.

## 4 Discussion

EWF provides a robust, efficient and exact sampling routine to target a wide family of Wright–Fisher diffusions featuring a broad class of selective regimes, any mutation parameters and any start/end points. The implementation can be used as a stand-alone package or incorporated into simulation-based inference pipelines from time series allele frequency data. This is particularly useful in view of the recent increase in availability of such data (Fages *et al.*, 2019; Wutke *et al.*, 2016).

## Supplementary Material

btad017_Supplementary_Data
